# Humoral immune response in children's patients with recurrent infections

**DOI:** 10.1186/1939-4551-8-S1-A218

**Published:** 2015-04-08

**Authors:** Valéria Soraya De Farias Sales, Vera Maria Dantas, Cléia Teixeira Amaral, Luanda Bárbara Ferreira Canário Souza, Raissa Anielle Silva Brandão, Geraldo Barroso Cavalcanti Júnior, Gabriela Vasconcelos De Andrade Alves, Sarah Dantas Viana Medeiros

**Affiliations:** 1 Federal University of Rio Grande Do Norte, Brazil

## Background

Primary immunodeficiency diseases comprise heterogeneous group of disorders that affect distinct components of the innate and adaptive immune system. The aim of this study was to evaluate the humoral response in children’s patients with recurrent infections.

## Methods

Venous blood samples were collected from 64 children in Pediatric Hospital Professor Heriberto Ferreira Bezerra (HOSPED) - Federal University of Rio Grande do Norte (UFRN), Brazil. The laboratory investigation included measurement of serum IgG, IgM and IgA levels by imunoturbidimetry and the B lymphocyte quantification by flow cytometry with monoclonal anti-CD19^+^ for the cases that presented decreased of the serum immunoglobulins.

## Results

Among the 64 children included, 33 (51,6%) were male and 31 (48,4%) were female. The children age ranged from 2 months to 15 years old. According to age, 36 (56%) children were aged 0-5 years; 11 (17%) children were aged 5 years and 1 month to 10 years; 10 (16%) children were aged 10 years and 1 month to 13 years and 11 months and 7 (11%) children were aged 14 years to 15 years. Immunoglobulin concentration below age-appropriate reference values was observed for IgG in 7 (11%) children; for IgM in 3 (4,7%) children and for IgA in 3 (4,7%) children. B lymphocytes were absent in 3 (4,7%) children. The superior respiratory tract infections were the most prevalent in this population.

## Conclusions

B-cell disorders are the most common type of immunodeficiencies and they are characterized by an increased susceptibility to respiratory tract infections. In this study, 3 patients received the clinical and laboratory diagnostic of X-linked agammaglobulinemia.

